# Integrating Landscape Metrics and Hydrologic Modeling to Assess the Impact of Natural Disturbances on Ecohydrological Processes in the Chenyulan Watershed, Taiwan

**DOI:** 10.3390/ijerph16020266

**Published:** 2019-01-18

**Authors:** Li-Chi Chiang, Yi-Ting Chuang, Chin-Chuan Han

**Affiliations:** 1 Department of Civil and Disaster Prevention Engineering, National United University, Miaoli City 36063, Taiwan; s946628a@gmail.com; 2 Department of Computer Science and Information Engineering, National United University, Miaoli City 36063, Taiwan; cchan@gm.nuu.edu.tw

**Keywords:** image classification, land cover change, landscape metrics, SWAT, watershed management

## Abstract

The Chenyulan watershed, located in the central mountain area of Taiwan, has been suffering from earthquakes, typhoons, and heavy rainfalls in recent decades. These sequential natural disturbances have a cumulative impact on the watershed, leading to more fragile and fragmented land cover and loss of capacity of soil water conservation. In this study, the Soil and Water Assessment Tool (SWAT) and a landscape metrics tool (FRAGSTATS) were used to assess the direct impact (e.g., by annual rainfall) and indirect impact (e.g., by landscape configuration and composition) of natural disturbances on the ecohydrological processes of the Chenyulan watershed. Six SPOT satellite images from 2008 to 2013 were analyzed by using the nearest feature line embedding (NFLE) approach and reclassified into six land cover types: forest, cultivated land, grassland, river, landslide, and built-up. Forest was found to have the largest patch size, indicating that it is more resilient to disturbances, while agricultural land tended to expand from the river side toward the hill. Two land cover change scenarios were compared in the SWAT model. The results showed that there was no significant difference in simulated streamflow during 2004–2015 and sediment loading during 2004–2009; however, the model performed better for sediment loading during 2010–2015 with dynamic land cover change (coefficient of determination (R^2^) = 0.66, Nash-Sutcliffe efficiency coefficient (NSE) = 0.62, percent bias (PBIAS) = 10.5%, root mean square error observation standard deviation ratio (RSR) = 0.62) than with constant land cover (R^2^ = 0.61, NSE = 0.54, PBIAS = −17.3%, RSR = 0.68), indicating that long-term land cover change should be considered in hydrologic modeling. Changes in landslides during 2008–2013 were found to significantly affect ecohydrological processes, especially after 2011. In general, annual precipitation plays a dominant role, and landscape composition had by far the strongest influence on water yield and sediment yield compared to landscape configuration. The results can be useful for understanding the effects of land cover change on ecohydrological processes in the Chenyulan watershed and the potential impact of ecohydrological changes on the environment and public health.

## 1. Introduction

Land use and land cover are the results of interactions among the natural environment and human activities, and their distribution can reflect anthropogenic types and decision behaviors [1]. Many studies have demonstrated the impact of land use and land cover change on streamflow, sediment exports, and other non-point source pollution loads at various spatiotemporal scales [2,3,4,5,6,7], and there is a need to identify the relationship between landscape metrics at the class scale and hydrological processes [8]. Many metrics and indices have been developed to characterize landscape compositions and spatial configurations on categorical maps [9,10]. These metrics are applied to quantify landscape changes over time [11,12], serve as landscape indicators [13,14,15,16], suggest strategies for watershed management [17], and investigate the relationship between landscape patterns and ecological processes [18].

The Soil and Water Assessment Tool (SWAT), a semi-distributed hydrologic model, is mainly used to simulate the impact of agricultural management on water quantity and quality in a watershed [19]. SWAT has been applied for hydrologic assessment [20,21,22,23], the evaluation of impacts of climate and land use change on streamflow [24,25] and water quality [26], modeling of ecosystem services [27,28], and best management practices (BMP) assessment [29,30]. In previous studies, surface runoff was found to be affected by landscape configuration and structure [31]. An increase in the connectivity between urban and agricultural lands could result in increased surface runoff [32]. Therefore, there is a need for a quantitative analysis of how landscape changes influence watershed streamflow and sediment export using hydrological models, in order to provide support for identifying the critical areas that require appropriate management and suggest strategies for future land use management and allocation. Moreover, based on interactions among ecohydrological factors and landscape configurations, ecohydrological factors that influence floods and landslides could be recognized to identify effective management practices to reduce their impact on the environment and public health.

Jhuoshuei River is the longest river in Taiwan, and Chenyulan River is its longest tributary. In recent years, due to inappropriate land use, the Chenyulan watershed has been suffering from floods, landslides, and debris flows generated by typhoons and heavy rainfall events [33]. As these natural disturbances have a cumulative impact on the landscape cover and watershed response, it is necessary to assess the change in ecohydrological processes. In this study, we collected and classified SPOT satellite images from 2008–2013 and analyzed the changes in spatial patterns by using FRAGSTATS (University of Massachusetts, Amherst, MA, USA) [34], which can quantify the landscape structure (i.e., composition and configuration). SWAT was applied to evaluate the impact of land cover changes during 2008–2013 on watershed responses in terms of water yield and sediment yield from different land cover types, and streamflow and sediment loading at the watershed outlet. Furthermore, we established a relationship between landscape metrics, water yield, and sediment yield. Such an integrated approach and improved understanding of this relationship would be useful for land use planners to reduce the risk of disasters and increase the ecosystem resilience of the watershed.

## 2. Materials and Methods

### 2.1. Study Area

**Figure 1:**
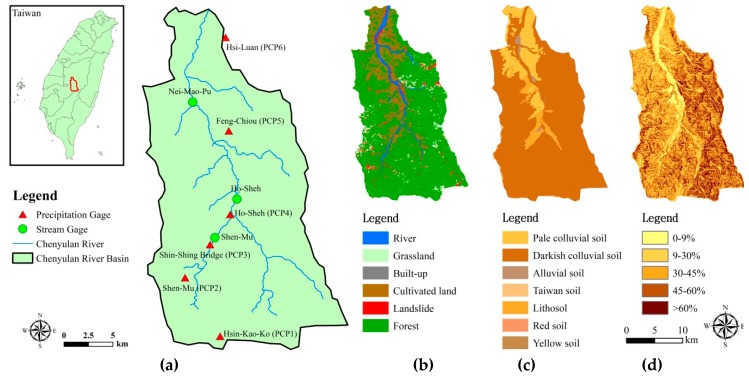
Observation stations and environmental information of the Chenyulan watershed: (**a**) locations of precipitation and stream gauges; (**b**) land use/cover; (**c**) soil; (**d**) slope.

The Chenyulan River, located in Nantou County, Taiwan, is 42 km long and originates from Mt. Jade at a height of 3910 m above sea level. The Chenyulan watershed has an area of 449 km^2^ elongated in the north-south direction, with an average altitude of 1540 m and a slope of 32° (Figure 1a). The observation stations include one weather station (namely PCP2 in this study), five automated precipitation gauges (PCP1, PCP3, PCP4, PCP5, PCP6), and three gauges of streamflow and sediment export (Shen-Mu, Ho-Sheh, Nei-Mao-Pu). Two gauges (Shen-Mu, Ho-Sheh) stopped recording streamflow and sediment export in 2001, and only Nei-Mao-Pu station has continuous records until now. More than 70% of the watershed is covered by forest, and cultivated lands are mostly distributed in the valley region (Figure 1b). Darkish colluvial soil is dominant (82.38%) in the watershed, followed by pale colluvial soil (12.29%), lithosol (4.19%), alluvial soil (0.89%), Taiwan clay (0.22%), yellow soil (0.03%), and red soil (0.002%) (Figure 1c). The major area (49.58%) is of a slope greater than 60%, followed by a slope of 45–60% (19.60%), 30–45% (15.14%), 9–30% (12.38%), and 0–9% (3.30%) (Figure 1d). The average annual precipitation in the Chenyulan watershed is between 2000 and 4000 mm, and approximately 80% of annual rainfall is between May and October (typhoon season). During 2008–2013, there were five to eight typhoons each year, and the most severe were Typhoon Sinlaku (1468.5 mm of accumulated rainfall at Shen-Mu gauge during 11–16 September 2008), Typhoon Morakot (2858.5 mm during 5–10 August 2009), Typhoon Fanapi (287.1 mm during 17–20 September 2010), Typhoon Nanmadol (241 mm during 27–31 August 2011), Typhoon Saola (665.5 mm during 30 July to 3 August 2012), and Typhoon Soulik (881 mm during 11–13 July 2013).

### 2.2. Data Source and Description

The key input data for hydrological modeling are the digital elevation model (DEM), soil data, weather data, and land use/cover data. The DEM data are at a 30 m resolution, processed by the Center for GIS, Research Center for Humanities and Social Sciences (RCHSS), Academia Sinica, Taiwan, in 2012. The soil data were collected from the Construction and Planning Agency, Ministry of the Interior, Taiwan. The surveyed soil data contain the soil erodibility factor (USLE-K); hydraulic conductivity; and percentages of silt, clay, and sand, while other information needed by the SWAT model (soil bulk density (SOL_BD), available water capacity of the soil layer (SOL_AWC), and saturated hydraulic conductivity (SOL_K)) were further calculated by using the soil-plant-air-water (SPAW) model developed by [35]. The basic soil parameters for the SWAT model are shown in Table 1. 

Daily weather parameters (precipitation, minimum and maximum air temperature, relative humidity, solar radiation, and wind speed) were collected from the Data Bank of Atmospheric and Hydrologic Research (DBAR), Taiwan. Daily streamflow and sediment loading were collected from the hydrological yearbook of Taiwan from 2003 to 2015, published by the Water Resources Agency, Ministry of Economic Affairs. During the study period, the average daily streamflow and sediment export were 26.34 cms and 4015.2 tons/day, respectively. High flow and high sediment loading are usually found during May and October. A severe typhoon, Morakot, generated 531–1134.5 mm of rainfall during 2–13 August 2009, resulting in the highest daily streamflow of 1555.81 cm and highest sediment loading of 5,535,292.91 tons/day. 

**Figure 2:**
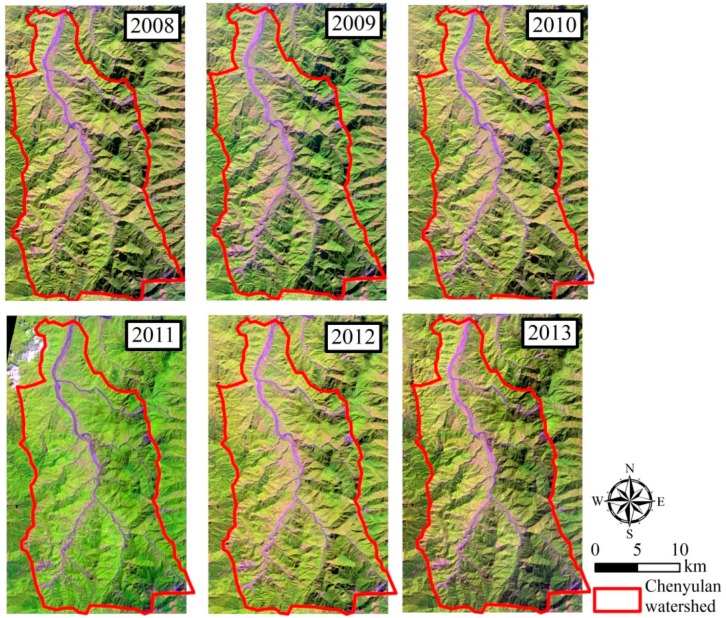
SPOT images during 2008–2013.

In order to evaluate the impact of natural disturbances on the watershed, we collected SPOT images taken on 28 November 2008, 2 December 2009, 21 November 2010, 22 September 2011, 25 October 2012, and 15 November 2013 (Figure 2). The SPOT images were purchased from the Space and Remote Sensing Research Center (SRSRC), and further used for watershed land cover classification.

### 2.3. Image Processing

#### 2.3.1. Selection of Ground-Truth Points

In order to select the ground-truth points for land cover classification, we first calculated the normalized difference vegetation index (NDVI) (Equation (1)). NDVI values range from –1 to +1. Negative values are mainly generated from clouds, water, and snow. A zero value means no vegetation (i.e., rocks and bare soil), and very low positive values (0.1 and below) represent barren areas. Moderate values (0.2–0.3) correspond to shrub and grassland, while values close to +1 indicate the highest possible density of green leaves (i.e., forests).

NDVI = (NIR − R)/(NIR + R),(1)

where NIR denotes near infrared reflectance and R denotes red (visible) reflectance.

**Figure 3:**
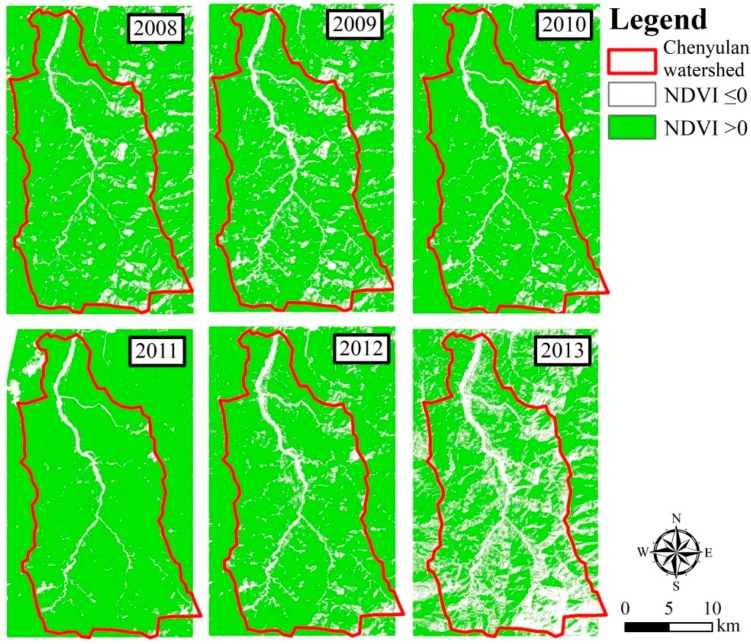
Normalized difference vegetation index (NDVI) values for images during 2008–2013.

Based on the NDVI maps (Figure 3), the watershed was divided into two groups: NDVI ≤ 0 and NDVI > 0. The areas of NDVI values smaller than 0 indicate that the possible land cover type is river, built-up, or landslide, while the areas of NDVI values greater than 0 indicate grassland, cultivated land, or forest. We additionally collected the land cover classifications of 1996 and 2005 from [33] and the landslide maps reclassified by satellite images derived from the Forestry Bureau, Council of Agriculture, Executive Yuan (FBCAEY) as reference maps. Areas where specific land classes were unchanged between 1996 and 2005 helped to narrow down the supervised boundary, and the FBCAEY landslide map helped to increase the accuracy of landslide area selection. 

**Figure 4:**
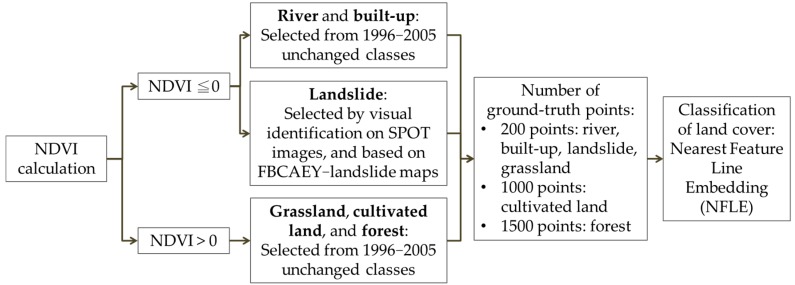
SPOT image processing procedure.

A number of points for each land cover type were selected based on the NDVI and reference maps (Figure 4). For example, 200 points were selected as water within the area of NDVI ≤ 0 in the NDVI map with reference to the water area in the unchanged 1996–2005 map. According to the relative sizes of land cover types, 200 points were selected for built-up, landslide, and grassland, and 1000 and 1500 points were selected for cultivated land and forest, respectively. 

#### 2.3.2. Nearest Feature Line Embedding

In our previous study [36], we proposed a nearest feature line (NFL) embedding transformation for land cover classification. The feature points (prototypes) were manually collected and labelled for classifier training. Since there are very few collected feature points during the training phase, the discriminant power of the trained classifier is decreased in the classification phase. A feature line is generated by two feature points and represents a linear interpolation or extrapolation of each pair of feature points within the same class. An extremely high number of pseudo-prototypes for each class are generated for training by linear interpolation, which enhances the classification performance. The NFL embedding strategy was used to construct the point-to-line adjacency matrix instead of the point-to-point matrix during training. This measurement was directly embedded in the transformation in the discriminant analysis, not in the classification phase. Class separability, neighborhood structure preservation, and nearest feature space (NFS) measurement were all considered to find the most effective and discriminating transformation in the eigenspaces for land cover classification. In this study, the images from 2008 to 2013 consist of four bands: green, red, near infrared reflectance (NIR), and shortwave infrared (SWIR). The criterion used for judging the accuracy of final SPOT images was an overall accuracy value exceeding 70%.

### 2.4. Landscape Metrics

Landscape metrics are usually used to describe the landscape ecosystem, format, and trend of landscape change to analyze the interactions among land uses and anthropogenic activities in watersheds [9,10]. We adopted FRAGSTATS software, developed by the United States Department of Agriculture (USDA), to quantify the composition and spatial configuration of land cover types [34]. Based on previous studies [37,38,39,40,41], we selected a subset of metrics that are commonly used and can affect ecohydrological processes to analyze landscape changes in the Chenyulan watershed from 2008 to 2013, and further evaluate how watershed responses and ecohydrological processes were affected by these changes. Landscape composition was quantified by the proportion of each land cover type. Configuration metrics included: (1) patch-based metrics: patch density (PD) and area-weighted mean patch area (AREA_AM); (2) shape metrics: edge density (ED), area-weighted mean radius of gyration (GYRATE_AM), and area-weighted mean shape index (SHAPE_AM); and (3) aggregation metrics: aggregation index (AI) and splitting index (SPLIT). The criteria for the landscape metrics were suggested by cases. Both PD and SPLIT describe the degree of subdivision of the class or landscape, and can be regarded as the degree of spread of disturbance in this study. AREA_AM, ED, and GYRATE_AM represent the physical continuity of the landscape, and can indirectly explain the influences on ecohydrologcial change. SHAPE measures the complexity of patch shape compared to a standard shape (square) of the same size. Thus, the index equals 1 for square patches of any size. AI refers to the tendency of patch types to be spatially aggregated. Detailed descriptions and equations of landscape metrics can be found in the FRAGSTATS documentation [34]. 

### 2.5. SWAT Model

#### 2.5.1. Model Description

The Soil and Water Assessment Tool (SWAT) was used to evaluate watershed responses to landscape changes induced by natural disturbances and anthropogenic activities in the Chenyulan watershed. The SWAT model was developed by the USDA Agricultural Research Service (USDA-ARS) in 1994, and it can predict long-term impacts of land use management on streamflow, sediment, and nutrient loadings in a watershed at different spatiotemporal scales [19]. In the SWAT model, a watershed is delineated into several subwatersheds, which are further portioned into homogeneous units (hydrologic response units, HRUs) with a unique combination of land use/cover, soil, and slope. For streamflow simulation, the surface runoff volume is computed using a modified Soil Conservation Service (SCS) curve number method [42]. The modified universal soil loss equation (MUSLE) was used to estimate soil loss at the HRUs [43]. More details on the theory can be found in the SWAT 2009 Theoretical Documentation [44]. 

#### 2.5.2. Land Cover Update Module

In order to incorporate land cover changes during 2008–2013 into the SWAT model, the land cover update (LUP) module in SWAT was activated. Two land cover scenarios were simulated to quantify the impact of land cover changes on water yield and sediment yield. They are constant land cover (CLC), which assumes that land cover remains constant since 2005, and updated land cover (ULC), which represents the dynamic land cover during 2008–2013. To activate the LUP module, two types of files need to be prepared. One is an lup.dat file, which lists the order of changing dates of each land cover, and the other is the HRU fraction (HRU_FR) file of different land covers of concern. For the ULC scenario, the SWAT model starts to read the land cover data on the date when the SPOT image was taken, and stops reading on the previous date before the next SPOT image was taken. 

#### 2.5.3. Model Calibration and Validation

The sensitivity analysis, calibration, and validation of the SWAT model were done by using the SWAT Calibration Uncertainty Program (SWAT-CUP), which is open source software developed by [45]. In this study, Sequential Uncertainty Fitting version 2 (SUFI2) was selected for uncertainty analysis. The model performance was evaluated by using four statistical measures: coefficient of determination (R^2^), Nash-Sutcliffe efficiency coefficient (NSE), percent bias (PBIAS), and root mean square error (RMSE)—observation standard deviation ratio (RSR), as suggested by [46].

## 3. Results

### 3.1. Classification Results

**Figure 5:**
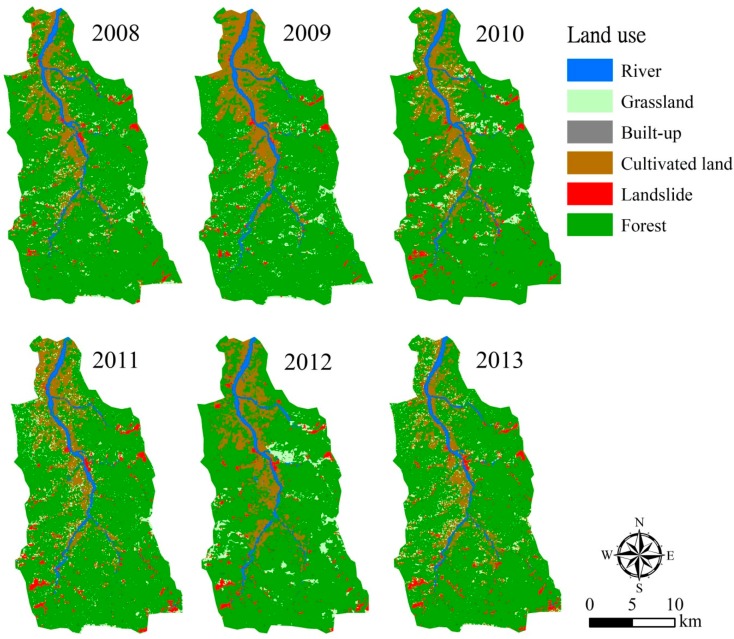
Multitemporal (2008–2013) classification results in the Chenyulan watershed.

The SPOT images were classified into six land cover types (river, grassland, built-up, cultivated land, landslide, and forest) (Figure 5 and Table 2), with the overall classification accuracy ranging between 73.70% and 83.74% (Table 3). Forest was the major land cover, occupying 74.45–76.75% of the watershed. Cultivated lands are usually developed along streams, with an area between 11.87% and 14.05% of the watershed. However, cultivated lands tended to be smaller and aggregated during the study period. The image classification results showed that river, grassland, and built-up areas did not change much during 2008–2013, with ranges of 2.97–3.63%, 4.34–5.72%, and 0.44–0.76%, respectively. Due to several severe typhoons during 2008–2009 and the cumulative impact of typhoons, landslides increased from 2.00% in 2008 to 2.73–3.11% during 2010–2013. 

It should be noted that the land cover data for 2008–2013 were adjusted based on the 2005 HRUs for updated land cover (ULC) scenario modeling. Compared to the original land cover areas, the adjusted land cover during 2008–2013 changed by −0.01 to 2.27 km^2^, −7.94 to 0.13 km^2^, −0.37 to 0.13 km^2^, 1.25–4.14 km^2^, 0.01–0.80 km^2^, and −4.99 to 4.30 km^2^ for water, grassland, built-up, cultivated, landslide, and forest, respectively (Table 2). In particular, grassland in 2005 was not evenly distributed in all subwatersheds. If grassland was identified in a subwatershed in any year during 2008–2013 where there was no grassland HRU in 2005, the grassland area would be replaced by cultivated land and forest. Therefore, the adjusted grassland area was generally smaller than the original area, while cultivated land and forest areas increased slightly after adjustment.

### 3.2. Landscape Metrics Analysis

#### 3.2.1. Landscape Level

Table 4 shows the Pearson’s correlation for seven landscape metrics at the landscape level, and their values for 2008–2013 are shown in Table 5. There is a strong positive relationship between PD and ED, with more patches and longer edge lengths, indicating a high level of fragmentation. SHAPE_AM was found to be positively correlated with PD and ED. A high SHAPE_AM value indicates that patch shapes are less compacted. GYRATE is equal to the mean distance between each cell and the centroid of that patch. GYRATE has a zero value when the patch consists of only one pixel, and increases without limit as the patch grows. Therefore, GYRATE_AM is sensitive to patch area (AREA_AM). SPLIT is negatively correlated with GYRATE_AM and AREA_AM, while AI is negatively correlated with PD and SHAPE_AM. 

A dramatic change was found in PD, AREA_AM, ED, and GYRATE_AM for the period 2010–2012 (Table 5). PD and ED values increased in 2011 and then dropped in 2012, while AREA_AM and GYRATE_AM values decreased in 2011 and then increased in 2012. Both grassland and forest are non–human-dominated green land cover types in this study; in particular, the change in forest dominates the change in landscape metrics. During 2010, 2011, and 2012, the grassland PD value was 1.06, 3.00, and 0.53 N/100 ha, and the grassland ED value was 10.44, 18.80, and 7.60 m/ha, respectively. In the same time period, the grassland AREA_AM value was 24.81, 6.52, and 91.51 m^2^, and the GYRATE_AM value was 215.63, 109.80, and 417.39 m, respectively. Forest PD and ED values were 0.49, 0.55, and 0.37 N/100 ha, and 27.37, 36.27, 29.49 m/ha, respectively, while AREA_AM and GYRATE_AM values were 33,154.72, 31,302.18, and 33,558.53 m^2^, and 8727.52, 8221.91, and 8766.38 m, respectively. Additionally, annual precipitation from 2010–2012 was 2206, 2098, and 3130 mm, respectively. Therefore, rainfall could influence grassland and forest configuration in the watershed. This finding is consistent with studies indicating that increased rainfall results in a higher percentage of shrub patches, with a higher shrub density and height [47,48]. Moreover, it was found that the GYRATE_AM value increased during 2008–2013, except in 2011, showing the process of fragmentation of a land cover patch beginning with a reduction in patch area and an increase in the proportion of edge-influenced patch area [49].

#### 3.2.2. Class Level

Table 6 shows the values of class-level landscape metrics and their Pearson’s correlations (Table 7). Forest occupies more than 70% of the watershed and usually exhibits as clusters. It is expected that forest has a low PD; the lowest SPLIT; and the highest AREA_AM, ED, GYRATE_AM, SHAPE_AM, and AI. As built-up is the smallest land cover type in the watershed, it has the smallest AREA_AM, ED, and GYRATE_AM. A small AI and high SPLIT indicate that built-up has very low connectivity. 

Generally, ED is positively correlated with PD for all land cover types, except forest. Similar to what is found in landscape-level results (Table 4), SPLIT is negatively correlated with GYRATE_AM and AREA_AM for all land cover types, as there is a strong positive correlation between GYRATE_AM and AREA_AM (Table 7). Moreover, some relationships are not found at the landscape level, but are significant at the class level, except forest (positive relationship among SHAPE_AM, AREA_AM, and GYRATE_AM, and negative relationship between SPLIT and SHAPE_AM). Forest is the major land cover type of the Chenyulan watershed, thus the uncorrelated relationships between SHAPE_AM and AREA_AM, between SHAPE_AM and GYRATE_AM, and between SPLIT and SHAPE_AM for forest could significantly affect these relationships at the landscape level. However, the relationship between PD and SHAPE_AM is not consistent at the landscape and class levels. While PD was positively correlated with SHAPE_AM at the landscape level, the relationship between PD and SHAPE_AM was positive for river and landslide, and was negative for grassland, built-up, and cultivated land.

### 3.3. Swat Results

#### 3.3.1. Model Calibration and Validation

**Figure 6:**
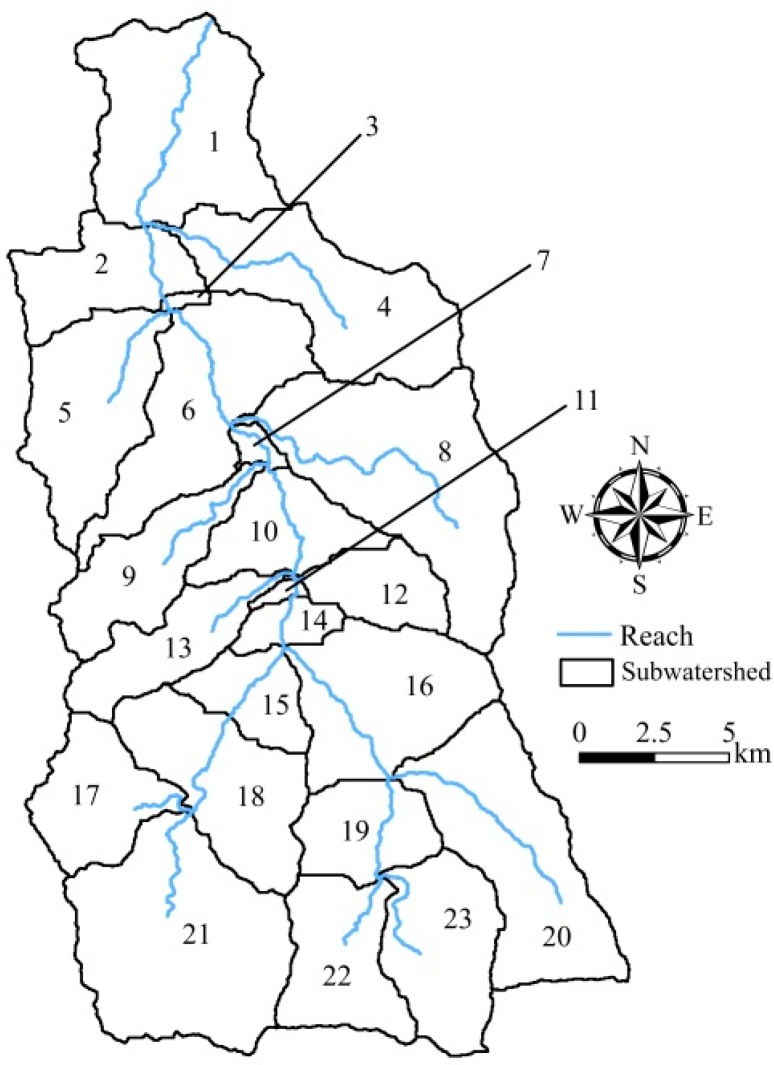
Distribution of 23 subwatersheds in the Chenyulan watershed.

SWAT was used to simulate the streamflow and sediment loading for constant land cover (CLC) and updated land cover (ULC) scenarios during 2003–2015. We selected 2003 as the warmup year, and 2004–2009 and 2010–2015 as the calibration and validation periods, respectively. A number of model parameters suggested by [50,51,52] were first examined for the sensitivity analysis. For streamflow parameters, a total of seven parameters with a *p*-value < 0.05 were selected for calibration. They were curve number (CN2), plant uptake compensation factor (EPCO), surface runoff lag time (SURLAG), baseflow alpha factor (ALPHA_BF), effective hydraulic conductivity in main channel alluvium (CH_K2), and Manning’s “*n*” value for the main channel (CH_N2). In order to reflect the heterogeneity of parameters at different locations in the watershed, some parameters were calibrated separately for head streams (subwatershed nos. 17, 20–23), subwatersheds at a slope greater than 60% (nos. 4, 8, 12, 16, 18, 19), and downstream subwatersheds (nos. 1–3, 5–7, 9–11, 13–15) (Figure 6). Table 8 lists the model parameters along with their default values, calibrated ranges, and fitted values. Details of the model parameters and their functions can be found in the SWAT 2012 Input/Output documentation [53].

CN2, which governs the surface runoff response, was calibrated for three land covers: forest (FRST), grassland (RNGE), and cultivated land (AGRL). The adjusted CN2 values indicate that the SWAT model with default parameters overestimated the daily streamflow. EPCO ranging from 0.01 to 1.00 was also calibrated in other studies [54,55]. When EPCO = 1.00, the model allows more of the water uptake demand to be met by lower layers in the soil. Therefore, a reduced EPCO indicates that the model allows less variation from the original depth distribution to take place. SURLAG controls the fraction of total available water that will be allowed to enter the reach on any one day [53]. As SURLAG increases, the streamflow hydrograph simulated in the reach will be smoother due to the delay in the release of surface runoff. ALPHA_BF can reflect the groundwater flow response to changes in recharge. A high value of CH_K2 indicates that the bed material has a very high loss rate and the stream is characterized as a flow-through stream that simultaneously receives and loses groundwater. As the head streams have more condense vegetation in the watershed, the CH_N2 value was calibrated higher than other reaches.

**Figure 7:**
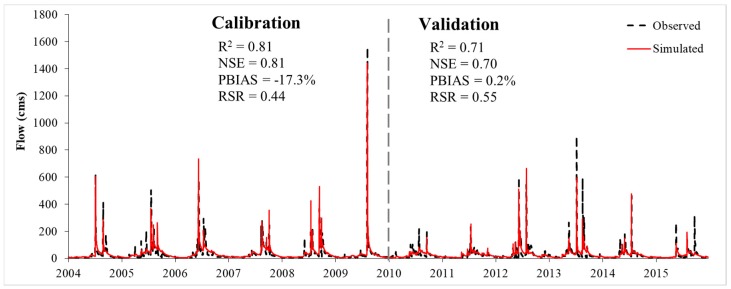
Comparison of simulated and observed daily streamflow during 2004–2015 at the Nei-Mou-Pu station.

The calibration and validation results for daily streamflow showed that there was no significant difference between two land cover scenarios. The model performance was very good in terms of R^2^ = 0.81, NSE = 0.81, PBIAS = −17.3%, and RSR = 0.44 for calibration, and R^2^ = 0.71, NSE = 0.7, PBIAS = 0.2%, and RSR = 0.55 for validation (Figure 7). 

**Figure 8:**
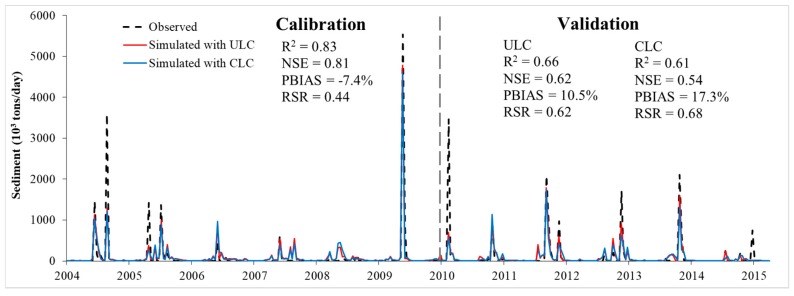
Comparison of simulated and observed daily sediment during 2004–2015 at the Nei-Mou-Pu station.

Two sediment-related parameters, the peak rate adjustment factor (PRF) for sediment routing in the main channel and the linear parameter for calculating the maximum amount of re-entrained sediment in the channel (SPCON), were calibrated for daily sediment prediction (Table 9). Both values increased, so the daily measured versus simulated sediment agreed well (Figure 8). For both land cover scenarios, the model performance was very good for calibration (R^2^ = 0.83, NSE = 0.81, PBIAS = −7.4%, and RSR = 0.44). Similar results from the calibration period (2004–2009) were mainly because the land cover started to change since 2008 in the ULU scenario and land cover change during 2008–2009 had little impact on the simulation. However, the model with the ULC scenario (R^2^ = 0.66, NSE = 0.62, PBIAS = 10.5%, and RSR = 0.62) performed better for validation than that with the CLC scenario (R^2^ = 0.61, NSE = 0.54, PBIAS = 17.3%, and RSR = 0.68), indicating that activating the LUP module in SWAT improved the model prediction.

#### 3.3.2. Swat Simulation Results

**Figure 9:**
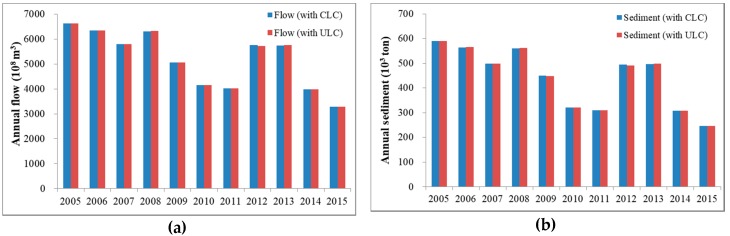
Comparison of simulation results under constant land cover (CLC) and updated land cover (ULC) scenarios during 2005–2015: (**a**) annual flow (m^3^); (**b**) annual sediment (tons).

Annual flow simulation was dominated by rainfall, leading to a similar trend of sediment loadings during 2005–2015 (Figure 9). For both land cover scenarios, annual flow ranged between approximately 3.3 × 10^11^ m^3^ and 6.6 × 10^11^ m^3^. Annual sediment loading ranged between 246,000 and 589,100 tons for the CLC scenario, and between 245,700 and 589,300 tons for the ULC scenario. The differences in annual flow and sediment between the two scenarios are mainly due to land cover changes and the yields from different land cover types (Table 10). Moreover, the impact of land cover change could be magnified by the rainfall. While built-up, landslide, and cultivated land decreased and forest increased in 2008, annual flow and sediment increased by 5 × 10^6^ m^3^ and 1900 tons under the ULC scenario, respectively. It should be noted that most of the SPOT images were taken late in the year, thus the impact of land use and land cover would be seen in the next year. Thus, the increase in annual flow and sediment under the ULC scenario could be the result of higher precipitation in 2008 magnifying the difference between 2005 and 2008 land covers. Since 2009, land cover change in the previous year had a greater impact in the simulation year than rainfall. Compared to the simulation of the CLC scenario in 2009, the amount of annual flow and sediment decreased under the ULC scenario due to a decrease in built-up, landslide, and cultivated land and an increase in forest in 2008. As for the cumulative impact of land cover change since 2008, the amount of annual flow and sediment decreased by 7.8 × 10^6^ m^3^ and 4000 tons in 2012 under the ULC scenario. 

## 4. Discussion

### 4.1. Impact of Land Cover Change on Ecohydrological Processes

**Figure 10:**
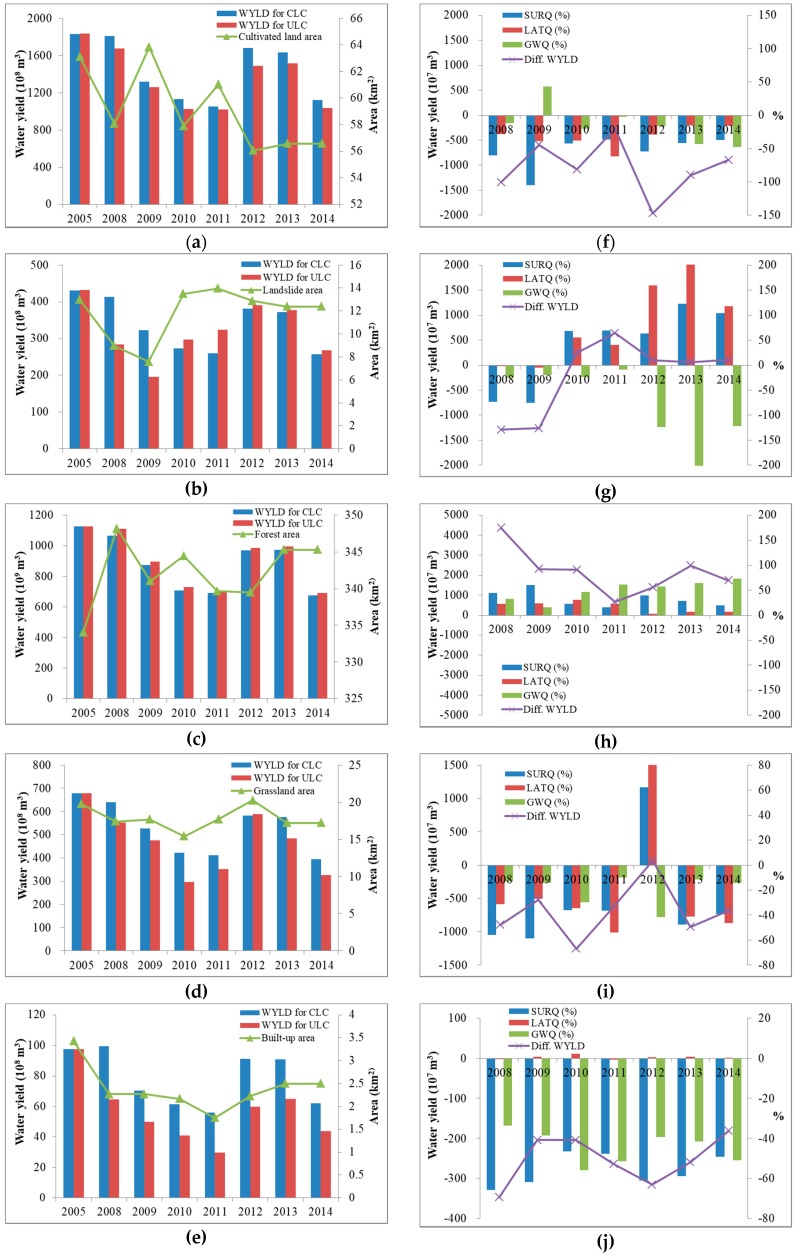
Annual water yield and composition under constant land cover (CLC) and updated land cover (ULC) scenarios: (**a**–**e**) water yield; (**f**–**j**) composition of water yield.

For the two scenarios, the annual water yields generated from different land covers (cultivated land, landslide, forest, grassland, and built-up) during 2005 and 2008–2014 were compared (Figure 10). The results showed that water yields were mainly affected by rainfall, so there was a similar trend of water yield generated and a similar average composition of water yield for all land cover types. For the constant land cover scenario, the water yield from cultivated land was composed of 50.92% surface runoff, 14.45% lateral flow, and 34.63% groundwater recharge; from forest, it was 34.69% surface runoff, 32.62% lateral flow, and 32.69% groundwater recharge; and from grassland, it was 44.30% surface runoff, 31.37% lateral flow, and 24.33% groundwater recharge. For the updated land cover scenario, the water yield from cultivated land was composed of 50.84% surface runoff, 13.46% lateral flow, and 35.70% groundwater recharge; from forest, it was 34.67% surface runoff, 32.32% lateral flow, and 33.00% groundwater recharge; and from grassland, it was 43.85% surface runoff, 30.63% lateral flow, and 25.51% groundwater recharge. However, land cover change had a slight impact on water yields generated from landslide and built-up. The composition of the water yield from landslide was 66.93% surface runoff, 13.52% lateral flow, and 19.55% groundwater recharge for the constant land cover scenario, and 67.72% surface runoff, 16.76% lateral flow, and 15.50% groundwater recharge for the updated land cover scenario. The contribution of lateral flow increased and groundwater recharge decreased for the land cover change scenario compared to the constant land cover scenario, indicating increasing pore water pressure, groundwater exfiltration from the bedrock, and hydraulic uplift pressure from below caused by landslides [56]. Similar changes in ecohydrological processes were found for built-up areas. The composition of water yield from built-up areas was 57.56% surface runoff, 4.46% lateral flow, and 37.98% groundwater recharge for the constant land cover scenario and 58.08% surface runoff, 6.74% lateral flow, and 35.17% groundwater recharge for the updated land cover scenario.

The difference in water yield was directly reflected by the change in land cover area during 2008–2014 (Figure 10f–j). Generally, due to decreasing areas of cultivated land, grassland, and built-up areas in the watershed, water yields decreased by 2.7 × 10^9^ to 19.6 × 10^9^ m^3^, 12.5 × 10^9^ to 5.2 × 10^9^ m^3^, and 3.5 × 10^9^ to 1.8 × 10^9^ m^3^, respectively, during 2008–2014 compared to the constant land cover scenario results. 

Landslides slightly increased after 2010, resulting in greater water yields for the updated land cover scenario (Figure 10g). However, the composition of the landslide water yield for both land cover scenarios changed. Surface runoff was the major contributor to the change in water yield during 2008–2011, while increasing lateral flow and decreasing groundwater recharge contributed during 2012–2014. The proportion of groundwater varied and the lateral flow surpassed surface runoff during 2012–2014. Moreover, the landslide areas similarly ranged between 12.39% and 13.97% for 2005 and 2010–2013. This shows that during the process of changing landslide proportion in the watershed over the years, the ecohydrological processes were altered. The variation in ecohydrological processes in landslide areas could be because the failures caused by landslide are mainly attributed to rapid transient variations in groundwater conditions [57]. 

Forest area was the smallest in 2005. Increasing forest area resulted in an increased water yield (Figure 10h). It was also found that the contribution of groundwater increased, indicating continuous groundwater recharge and replenishing rates. Built-up areas primarily consisting of impervious surfaces increase surface runoff and prevent groundwater from recharging to the land. Therefore, decreases in surface runoff (−46.45% to −65.80%) and groundwater recharge (−33.68% to −56.05%) were the two major contributors to the change in built-up water yield between land cover scenarios (Figure 10j).

### 4.2. Relationship between Landscape Metrics and Watershed Responses

Table 11 shows the interactions between water yield, sediment yield, and landscape metrics at the class level. It should be noted that subwatershed landscape metrics at the class level were individually calculated for each year from 2008–2013, and water yield and sediment yield were simulated under the updated land cover scenario. Because most of the SPOT images were taken in the second half of the year, under simulation, the landscape pattern shows the impact the following year. Thus, simulated watershed responses during 2009–2014 were compared with landscape metrics during 2008–2013. Although the SWAT model is a semi-distributed hydrological model and the simulation results are dominated by landscape composition rather than landscape configuration, the relationship between landscape metrics and watershed responses could be regarded as a cause-and-effect relationship between landscape composition and configuration. 

It was found that all built-up landscape metrics (configuration) were not significantly correlated with water yield and sediment yield, as built-up area is the smallest in the watershed. The patch density (PD) of grassland, cultivated land, and forest was negatively correlated with water yield, indicating that water yield could increase due to increasing greenland in amount and size. In a defined area, a higher patch density means smaller patch areas and more patch edges. The edge density (ED) of grassland, cultivated land, and forest was relatively higher (13.32 m, 21.20 m, 31.46 m, respectively) than the other land covers (built-up 1.81 m, landslide 5.22 m) (Table 7). The PD and ED analyses proved that the higher the patch and edge density of grassland, the lower the generation of water in cultivated land and forest. This result is in agreement with the finding that increasing agricultural patch density leads to a decrease in surface runoff and sediment yield [17]. 

Through the fragmentation process, landscape composition and spatial configuration are affected (e.g., patch area, number of patches, patch shape complexity, number of patch edges, distances between patches), resulting in a change in landscape connectivity [49]. Thus, the aggregation index [58] and splitting index [59] are usually used to assess this change. AI reflects the physical aggregation of land covers within a watershed, and a higher value means more aggregation [34]. At the class level, the AI metric was positively correlated with water yield from forest and sediment yield from landslide, while it had a negative relationship with water and sediment yield from cultivated land (Table 11). As baseflow decreases, an increase in surface runoff is induced by the increasing AI of forest [17]. Moreover, when the landscape is dominated (e.g., >60%) by a given cover type, it is nearly always well connected [60]. The different relationships between the AI metric and water/sediment yield indicate that more water and sediment are found when forest and landslide are more aggregated and cultivated lands are more scattered over the watershed. This finding also indicates that when those landscape areas expand, forest and landslide tend to be more aggregated and cultivated lands tend to be more scattered. Land cover patterns could affect ecohydrological processes and components of the water yield, but also control the amount of water and sediment yield within the watershed. Moreover, the shape metric (SHAPE_AM) of landslide had a positive trend with sediment yield, indicating that a large shape can intensify erosion [39].

The shape indices (ED, GYRATE_AM, and SHAPE_AM) of cultivated lands had negative relationships with water yield and sediment yield, while those indices of landslide had positive relationships with both yields. Usually, the flow rates between land cover types can be enhanced or disrupted, depending on the hardness of the edges [8]. Cultivated land that is disrupted by humans has straight and sharp edges [61], and natural landslides have more curvilinear borders. Thus, the edge characteristics may partially determine the erosion characteristics and sediment export [8]. Moreover, a lower aggregation index (AI) and higher splitting index (SPLIT) resulting in a greater water yield and sediment yield indicate that many small and interspersed land cover patches are more likely to accelerate soil erosion and increase sediment yields [8]. 

## 5. Conclusions

Landscape structures and patterns can affect runoff and non-point source pollution loadings in watersheds [58], and the impact of a single land cover can be affected by other land cover types at the watershed scale [39]. In this study, we first classified SPOT images into six land cover types by using the nearest feature line embedding (NFLE) method, and then quantified the landscape patterns by FRAGSTATS at the landscape and class levels. Our goals were to investigate how landscape patterns affect the water yield and sediment yield from different land cover types, and also to understand how ecohydrological processes are changed when updated land cover change is considered in the SWAT model. The results showed that SWAT could more accurately predict changes in streamflow and sediment exports under the updated land cover scenario. Although the SWAT model is a semi-distributed hydrological model, the relationship between landscape metrics and watershed responses could be regarded as a cause-and-effect relationship between landscape composition and configuration.

The indirect impact of natural disturbances was reflected in the change in landscape configuration, in terms of more fragmentation during 2009–2011 with increasing patch density (PD), edge density (ED), and area-weighted mean shape index (SHAPE_AM). Annual precipitation was the dominant influence on the amount of water yield, while the difference in water yield between land cover scenarios was led by the change in land cover area (landscape composition). The relationships between landscape metrics, water yield, and sediment yield were significant but with a relatively low statistical value, indicating that landscape composition had by far the stronger influence. This finding is in agreement with other studies [62,63,64]. Moreover, the shape indices (ED, GYRATE_AM, and SHAPE_AM) of cultivated lands had a negative relationship with water and sediment yield, while those indices of landslide had a positive relationship with water and sediment yield. However, [39] found that a proper fragile landscape status and more complicated patches can reduce the soil erosion yield and more patch edges can prevent soil erosion by disturbing formation and transportation; and [65] suggested that spatial distribution and the number of farmland areas need to be considered to reduce sediment yields. The contrasting results of previous studies and this study show the complicated relationship between landscape patterns and sediment yield.

By identifying the contributions of different hydrological components to water yield, we can understand how changes in land cover affect ecohydrological processes and how the ecohydrological changes could further affect the environment and public health. Therefore, landscape characteristics that can influence ecohydrological processes should be considered in watershed management, and landscape configuration at the subwatershed level should be considered in the SWAT model. The various interactions between class-level landscape metrics, water yield, and sediment yield are useful for providing guidelines on soil erosion prevention and sustainable hydrologic ecosystem services. 

## Figures and Tables

**Table 1:** Basic soil parameters in the Chenyulan watershed.

Soil Type	Hydrologic Group	USLE-K	SOL_BD (g/cm^3^)	SOL_AWC (mm H_2_O/mm soil)	SOL_K (mm/hr)
Darkish colluvial soils	B	0.36	1.54	1.54	25.91
Pale colluvial soil	B	0.19	1.49	0.14	9.65
Lithosol	B	0.3	1.56	0.13	4.83
Alluvial soil	B	0.4	1.61	0.17	7.87
Taiwan clay ^1^	B	0.2	1.43	0.15	2.03
Yellow soil	B	0.29	1.52	0.15	10.92
Red soil	B	0.13	1.60	0.19	21.34

^1^ The parent material is not seen in this type of soil, which is usually thick-layered and compacted, and has poor tillage due to poor drainage. USLE-K, soil erodibility factor; SOL_BD, soil bulk density; SOL_AWC, available water capacity of soil layer; SOL_K, saturated hydraulic conductivity.

**Table 2:** Land cover area (km^2^) in 2005 and 2008–2013.

Land Cover Type	2005	2008	2009	2010	2011	2012	2013
River	15.42	13.76 (13.75) ^1^	16.31 (16.29)	15.19 (15.20)	14.96 (14.60)	15.55 (17.82)	13.31 (13.39)
Grassland	19.77	20.21 (17.40)	19.47 (17.68)	19.62 (15.43)	25.67 (17.73)	20.12 (20.24)	23.81 (17.23)
Built-up	3.42	2.60 (2.27)	2.33 (2.27)	2.17 (2.17)	1.98 (1.75)	2.10 (2.23)	2.86 (2.50)
Cultivated land	63.07	56.55 (58.07)	62.58 (63.84)	55.31 (57.91)	56.87 (61.01)	54.05 (56.05)	53.27 (56.58)
Landslide	12.97	8.96 (8.97)	7.57 (7.57)	12.70 (13.50)	13.97 (13.97)	12.53 (12.87)	12.24 (12.39)
Forest	334.15	346.72 (348.20)	340.54 (331.02)	343.82 (344.46)	335.36 (339.66)	344.47 (339.48)	343.31 (345.27)

^1^ Numbers in parentheses are adjusted areas simulated for updated land cover scenario.

**Table 3:** Assessment index of classification accuracy.

Year	2008	2009	2010	2011	2012	2013
Overall accuracy	82.22%	83.74%	81.00%	81.81%	77.80%	73.70%

**Table 4:** Correlation matrix of landscape metrics at the landscape level. PD, patch density; AREA_AM, area-weighted mean patch area; ED, edge density; GYRATE_AM, area-weighted mean radius of gyration; SHAPE_AM, area-weighted mean shape index; AI, aggregation index; SPLIT, splitting index.

Metrics	PD	AREA_AM	ED	GYRATE_AM	SHAPE_AM	AI	SPLIT
PD	1						
AREA_AM	−0.319	1					
ED	0.968 **	−0.283	1				
GYRATE_AM	−0.396	0.931 **	−0.335	1			
SHAPE_AM	0.879 *	−0.036	0.930 **	−0.094	1		
AI	−0.969 **	0.282	−1.000 **	0.335	−0.930 **	1	
SPLIT	0.321	−1.000 **	0.286	−0.935 **	0.041	−0.285	1

** *p* < 0.01; * *p* < 0.05.

**Table 5:** Landscape-level metrics during 2008–2013.

Land Cover Type	2008	2009	2010	2011	2012	2013
PD	4.98	3.63	3.66	6.11	2.95	6.80
AREA_AM	25,331.01	24,734.56	25,550.06	23,471.01	25,895.42	25,801.08
ED	38.98	31.80	35.12	45.48	33.63	47.09
GYRATE_AM	6741.61	6780.94	6984.70	6527.08	7003.28	6970.06
SHAPE_AM	14.43	12.59	12.93	15.94	14.21	17.79
AI	80.39	84.03	82.36	77.14	83.12	76.31
SPLIT	1.79	1.83	1.77	1.92	1.74	1.75

**Table 6:** Average landscape metrics during 2008–2013 for all land covers.

Land Cover	PD	AREA_AM	ED	GYRATE_AM	SHAPE_AM	AI	SPLIT
Grassland	1.85	25.94	13.32	190.40	1.67	33.01	77,723.66
Built-up	0.37	2.39	1.81	66.75	1.10	12.88	3,900,140.18
Cultivated land	1.26	549.57	21.20	1255.08	5.87	57.51	1264.99
Landslide	0.54	26.01	5.22	223.12	1.60	49.15	81,111.31
Forest	0.44	32,791.92	31.46	8537.20	17.79	89.41	1.81

**Table 7:** Pearson’s correlation for seven landscape metrics at the class level.

Landscape Metrics		Land Cover					
		River	Grassland	Built-up	Cultivated Land	Landslide	Forest
**AREA_AM**	**PD**	0.575	−0.822 *	−0.417	−0.779	0.370	−0.705
**ED**	**PD**	0.927 **	0.993 **	0.975 **	0.972 **	0.935 **	−0.123
**ED**	**AREA_AM**	0.768	−0.806	−0.207	−0.802	0.628	−0.106
**GYRATE_AM**	**PD**	0.401	−0.885 *	−0.598	−0.771	0.462	−0.559
**GYRATE_AM**	**AREA_AM**	0.925 **	0.991 **	0.888 *	0.988 **	0.989 **	0.905 *
**GYRATE_AM**	**ED**	0.539	−0.867 *	−0.437	−0.761	0.701	0.171
**SHAPE_AM**	**PD**	0.694	−0.923 **	−0.534	−0.805	0.755	−0.285
**SHAPE_AM**	**AREA_AM**	0.823 *	0.955 **	0.850 *	0.905 *	0.808	0.137
**SHAPE_AM**	**ED**	0.736	−0.893 *	−0.377	−0.727	0.901 *	0.970 **
**SHAPE_AM**	**GYRATE_AM**	0.739	0.984 **	0.984 **	0.954 **	0.880 *	0.387
**AI**	**PD**	−0.523	−0.961 **	−0.032	−0.970 **	−0.385	0.047
**AI**	**AREA_AM**	−0.019	0.942 **	0.886 *	0.859 *	0.616	0.165
**AI**	**ED**	−0.278	−0.944 **	0.180	−0.991 **	−0.105	−0.997 **
**AI**	**GYRATE_AM**	0.022	0.978 **	0.603	0.827 *	0.519	−0.128
**AI**	**SHAPE_AM**	−0.568	0.989 **	0.548	0.796	0.070	−0.953 **
**SPLIT**	**PD**	−0.639	0.928 **	−0.128	0.849 *	−0.731	0.752
**SPLIT**	**AREA_AM**	−0.948 **	−0.765	−0.846 *	−0.662	−0.865 *	−0.984 **
**SPLIT**	**ED**	−0.851 *	0.929 **	−0.340	0.744	−0.894 *	0.207
**SPLIT**	**GYRATE_AM**	−0.806	−0.832 *	−0.643	−0.718	−0.904 *	−0.853 *
**SPLIT**	**SHAPE_AM**	−0.742	−0.864 *	−0.643	−0.853 *	−0.900 *	−0.029
**SPLIT**	**AI**	−0.051	−0.901 *	−0.932 **	−0.793	−0.324	−0.270

** *p* < 0.01; * *p* < 0.05.

**Table 8:** Calibrated parameters for streamflow. CN2, curve number; EPCO, uptake compensation factor; SURLAG, surface runoff lag time; ALPHA_BF, baseflow alpha factor; CH_K2, effective hydraulic conductivity in main channel alluvium; CH_N2, Manning’s “*n*” value for the main channel; FRST, forest; RNGE, grassland; AGRL, cultivated land.

Parameter	Unit	Default Value	Calibrated value		
			Min.	Max.	Fitted
CN2	-	60 (FRST)	35 (−41.94%)	57 (−4.66%)	37 (−38.68%)
		69 (RNGE)	39 (−44.10%)	61 (−12.05%)	39 (−43.38%)
		77 (AGRL)	43 (−44.10%)	68 (−12.05%)	44 (−43.38%)
EPCO	-	1	0.10	0.44	0.42
SURLAG	-	4	6.43	18.15	11.50
ALPHA_BF	1/days	0.048 (sub1–3, 5–7, 9–11, 13–15)	0.18	0.53	0.34
		0.048 (sub4, 8, 12, 16, 18, 19)	0	0.43	0.23
CH_K2	mm/hr	0 (sub1–3, 5–7, 9–11, 13–15)	342.85	555.75	510.51
		0 (sub4, 8, 12, 16, 18, 19)	333.03	583.73	571.82
		0 (sub17, 20–23)	293.20	579.25	439.80
CH_N2	-	0.014 (sub4, 8, 12, 16, 18, 19)	0.10	0.23	0.18
		0.014 (sub17, 20–23)	0.19	0.32	0.22

**Table 9:** Calibrated parameters for sediment. PRF, peak rate adjustment factor; SPCON, linear parameter for maximum re-entrained sediment.

Parameter	Unit	Default Value	Calibrated Value		
			Min.	Max.	Fitted
PRF	-	1.0	1.01	1.82	1.49
SPCON	-	0.00001	0.008	0.016	0.013

**Table 10:** Annual average water yield (WYLD) and sediment yield (SYLD) from different land covers under ULC scenario.

Land cover	Grassland	Built-up	Cultivated Land	Landslide	Forest	Average
WYLD (mm)	2381.49	2109.56	2102.97	2548.91	2432.68	2386.46
SYLD (tons/km^2^)	50,868.24	24,281.99	271,683.10	1,591,249.74	53,825.47	125,447.62

**Table 11:** Interaction between water yield, sediment yield, and landscape metrics at the class level.

**Water Yield**							
**Land Cover**	**PD**	**AREA_AM**	**ED**	**GYRATE_AM**	**SHAPE_AM**	**AI**	**SPLIT**
Grassland	−0.271 **	0.1025	−0.278 **	0.1007	0.0054	0.2052	−0.0382
Built-up	−0.1534	0.0599	−0.0848	0.0302	−0.0161	−0.0815	0.0613
Cultivated land	−0.207 *	−0.1702	−0.341 **	−0.270 **	−0.252 **	−0.421 **	0.375 **
Landslide	−0.0030	0.0158	0.0829	0.0558	0.1030	0.0625	−0.0625
Forest	−0.282 **	0.394 **	−0.350 **	0.353 **	0.0314	0.304 **	−0.1547
**Sediment Yield**							
**Land Cover**	**PD**	**AREA_AM**	**ED**	**GYRATE_AM**	**SHAPE_AM**	**AI**	**SPLIT**
Grassland	0.0240	0.0944	0.0622	0.1102	0.0613	0.0182	−0.1205
Built-up	−0.0805	−0.0328	−0.0836	−0.0018	0.0035	−0.0468	−0.0842
Cultivated land	−0.228 **	−0.0849	−0.377 **	−0.216 *	−0.223 *	−0.431 **	0.327 **
Landslide	0.1777	0.1796	0.349 **	0.281 **	0.341 **	0.238 *	−0.1639
Forest	−0.0156	−0.0541	0.213 *	0.0442	0.257 **	−0.0851	−0.0390

** *p* < 0.01; * *p* < 0.05.

## References

[1] Lambin E.F., Turner B.L., Geist H.J., Agbola S.B., Angelsen A., Bruce J.W., Coomes O.T., Dirzo R., Fischer G., Folke C. (2001). The causes of land-use and land-cover change: Moving beyond the myths. Glob. Environ. Chang..

[2] Fohrer N., Haverkamp S., Eckhardt K., Frede H.G. (2001). Hydrologic response to land use changes on the catchment scale. Phys. Chem. Earth (B).

[3] Pikounis M., Varanou E., Baltas E., Dassaklis A., Mimikou M. (2003). Application of the SWAT model in the Pinious River Basin under different land-use scenarios. Environ. Sci. Technol..

[4] Ghaffari G., Keesstra S., Ghodousi J., Ahmadi H. (2010). SWAT-simulated hydrological impact of land-use change in the Zanjanrood Basin, northwest Iran. Hydrol. Processes.

[5] Du J., Rui H., Zuo T., Li Q., Zheng D., Chen A., Xu Y., Xu C.Y. (2013). Hydrological simulation by SWAT model with fixed and varied parameterization approaches under land use change. Water Resources Manag..

[6] Wagner P.D., Kumar S., Schneider K. (2013). An assessment of land use change impacts on the water resources of the Mula and Mutha Rivers catchment upstream of Pune, India. Hydrol. Earth Syst. Sci..

[7] Zope P.E., Eldho T.I., Jothiparkash V. (2017). Hydrological impacts of land use—Land cover change and detention basins on urban flood hazard: A case study of Poisar River basin, Mumbai India. Nat. Hazards.

[8] Shi Z.H., Ai L., Li X., Huang X.D., Wu G.L., Liao W. (2013). Partial least-squares regression for linking land-cover patterns to soil erosion and sediment yield in watersheds. J. Hydrol..

[9] McGarigal K., Marks B.J. (1995). FRAGSTATS: A Spatial Pattern Analysis Program for Quantifying Landscape Structure.

[10] McGarigal K., Cushman S.A., Neel M.C., Ene E. (2002). FRAGSTATS: Spatial Pattern Analysis Program for Categorical Maps. http://www.umass.edu/landeco/research/fragstats/fragstats.html.

[11] O’Neill R.V., Hunsaker C.T., Jones K.B., Riitters K.H., Wickham J.D., Schwartz P.M., Goodman I.A., Jackson B.L., Baillargeon W.S. (1997). Monitoring environmental quality at the landscape scale. BioScience.

[12] Frohn R.C., Hao Y. (2006). Landscape metric performance in analyzing two decades of deforestation in the amazon basin of rondonia, Brazil. Remote Sens. Environ..

[13] Turner M.G. (2005). Landscape ecology: What is the state of the science?. Annu. Rev. Ecol. Evol. Syst..

[14] Uuemaa E., Roosaare J., Mander U. (2005). Scale dependence of landscape metrics and their indicatory value for nutrient and organic matter losses from catchments. Ecol. Indic..

[15] Uuemaa E., Roosaare J., Mander U. (2007). Landscape metrics as indicators of river water quality at catchment scale. Nord. Hydrol..

[16] Uuemaa E., Mander L., Marja R. (2013). Trends in the use of landscape spatial metrics as landscape indicators: A review. Ecol. Indic..

[17] Boongaling C.G., Faustino-Eslava D.V., Lansigan F.P. (2018). Modeling land use change impacts on hydrology and the use of landscape metrics as tools for watershed management: The case of an ungauged catchment in the Philippines. Land Use Policy.

[18] Wickham J.D., Riitters K.H., O’Neill R.V., Reckhow K.H., Wade T.G., Jones K.B. (2000). Land cover as a framework for assessing risk of water pollution. J. Am. Water Resour. Assoc..

[19] Arnold J.G., Srinivasan R., Muttiah R.S., Williams J.R. (1998). Large-area hydrologic modeling and assessment: Part I. Model development. J. Am. Water Resour. Assoc..

[20] Anand J., Gosain A.K., Khosa R., Srinivasan R. (2018). Regional scale hydrologic modeling for prediction of water balance, analysis of trends in streamflow and variations in streamflow: The case study of the Ganga River basin. J. Hydrol. Reg. Stud..

[21] Ndulue E.L., Ezenne G.I., Mbajiorgu C.C., Ogwo V. (2018). Hydrological modeling of upper Ebonyi watershed using the SWAT model. Int. J. Hydrol. Sci. Technol..

[22] Oeurng C., Cochrane T.A., Arias M.E., Shrestha B., Piman T. (2016). Assessment of changes in riverine nitrate in the Sesan, Srepok and Sekong tributaries of the lower Mekong River basin. J. Hydrol. Reg. Stud..

[23] Liu R., Wang Q., Xu F., Men C., Guo L. (2017). Impacts of manure application on SWAT model outputs in the Xiangxi River watershed. J. Hydrol..

[24] Ahiablame L., Sinha T., Paul M., Ji J.H., Rajib A. (2017). Streamflow response to potential land use and climate changes in the James River watershed, Upper Midwest United States. J. Hydrol. Reg. Stud..

[25] Guzha A.C., Rufino M.C., Okoth S., Jacobs S., Nóbrega R.L.B. (2017). Impacts of land use and land cover change on surface runoff, discharge and low flows: Evidence from East Africa. J. Hydrol. Reg. Stud..

[26] Wang Q., Liu R., Men C., Guo L. (2018). Application of genetic algorithm to land use optimization for non-point source pollution control based on CLUE-S and SWAT. J. Hydrol..

[27] Francesconi W., Srinivasan R., Pérez-Miñana E., Willcock S.P., Quintero M. (2016). Using the Soil and Water Assessment Tool (SWAT) to model ecosystem services: A systematic review. J. Hydrol..

[28] Sil A., Rodrigues A.P., Carvalho-Santos C., Nunes J.P., Honrado J., Alonso J., Marta-Pedroso C., Azevedo J.C. (2016). Trade-offs and synergies between provisioning and regulating ecosystem services in a mountain area in Portugal affected by landscape change. Mt. Res. Dev..

[29] Chen L., Wei G., Shen Z. (2016). Incorporating water quality responses into the framework of best management practices optimization. J. Hydrol..

[30] Park J.Y., Ale S., Teague W.R. (2017). Simulated water quality effects of alternate grazing management practices at the ranch and watershed scales. Ecol. Model..

[31] Kalantari Z., Lyon S.W., Folkeson L., French H.K., Stolte J., Jansson P.E., Sanssner M. (2014). Quantifying the hydrological impact of simulated changes in land use on peak discharge in a small catchment. Sci. Total Environ..

[32] Marhaento H., Booij M.B., Rientjes T.H.M., Hoekstra A.Y. Simulation of land use change impacts on hydrological processes in a tropical catchment. Proceedings of the 19th EGU General Assembly, EGU2017.

[33] Chiang L.C., Lin Y.P., Huang T., Schmeller D.S., Verburg P.H., Liu Y.L., Ding T.S. (2014). Simulation of ecosystem service responses to multiple disturbances from an earthquake and several typhoons. Landsc. Urban Plan..

[34] McGarigal K. (2014). FRAGSTATS Help. http://refhub.elsevier.com/S0048-9697(16)31293-1/rf0150.

[35] Saxton K., Willey P. (2005). The SPAW Model for agricultural field and pond hydrologic simulation. Watershed Models.

[36] Chang Y.L., Liu J.N., Han C.C., Chen Y.N. (2014). Hyperspectral image classification using nearest feature line embedding approach. IEEE Trans. Geosci. Remote Sens..

[37] Amiri B.J., Nakane K. (2009). Modeling the linkage between river water quality and landscape metrics in the Chugoku District of Japan. Water Resour. Manag..

[38] Lee S.W., Hwang S.J., Lee S.B., Hwang H.S., Sung H.C. (2009). Landscape ecological approach to relationships of land use patterns in watersheds to water quality characteristics. Landsc. Urban Plan..

[39] Ouyang W., Skidmore A.K., Hao F., Wang T. (2010). Soil erosion dynamics response to landscape pattern. Sci. Total Environ..

[40] Li J., Song C.S., Cao L., Zhu F., Meng X., Wu J. (2011). Impacts of landscape structure on surface urban heat islands: A case study of Shanghai, China. Remote Sens. Environ..

[41] Wu W., Zhao S., Zhu C., Jiang J. (2015). A comparative study of urban expansion in Beijing, Tianjin and Shijiazhuang over the past three decades. Landsc. Urban Plan..

[42] USDA Soil Conservation Service (1972). National Engineering Handbook Section 4 Hydrology, Chapters 4–10.

[43] Williams J.R. (1975). Sediment—Yield Prediction with Universal Equation Using Runoff Energy Factor. Proceedings of the Sediment-Yield Workshop.

[44] Neitsch S.L., Arnold J.G., Kiniry J.R., Williams J.R. (2011). Soil and Water Assessment Tool Theoretical Documentation Version 2009.

[45] Abbaspour K.C. (2015). SWAT-CUP: SWAT Calibration and Uncertainty Programs—A User Manual.

[46] Moriasi A., Arnold J.G., Van Liew M.W., Bingner R.L., Haemel R.D., Veith T.L. (2007). Modeling evaluation guidelines for systematic qualification of accuracy in watershed simulation. Am. Soc. Agric. Biol. Eng..

[47] Shackleton C.M., Scholes R.J. (2011). Above ground woody community attributes, biomass and carbon stocks along a rainfall gradient in the savannas of the central lowveld, South Africa. S. Afr. J. Bot..

[48] Fan Y., Li X.Y., Huang Y.M., Liu L., Zhang J.H., Liu Q., Jiang Z.Y. (2017). Shrub patch configuration in relation to precipitation and soil properties in Northwest China. Ecohydrology.

[49] Neel M.C., McGarigal K., Cushman S.A. (2004). Behavior of class-level landscape metrics across gradients of class aggregation and area. Landsc. Ecol..

[50] Abbaspour K.C., Yang J., Maximov I., Siber R., Bogner K., Mieleitner J., Zobrist J., Srinivasan R. (2007). Modelling hydrology and water quality in the pre-alpine/alpine Thur watershed using SWAT. J. Hydrol..

[51] Abbaspour K.C., Rouholahnejad E., Vaghefi S., Srinivasan R., Yang H., Klove B. (2015). A continental-scale hydrology and water quality model for Europe: Calibration and uncertainty of a high-resolution large-scale SWAT model. J. Hydrol..

[52] Arnold J.G., Moriasi D.N., Gassman P.W., Abbaspour K.C., White M.J., Srinivasan R., Santhi C., Harmel R.D., van Griensven A., Van Liew M.W. (2012). SWAT: Model use, calibration, and validation. Trans. ASABE.

[53] Arnold J.G., Kiniry J.R., Srinivasan R., Williams J.R., Haney E.B., Neitsch S.L. (2012). SWAT Input/Output Documentation Version 2012.

[54] Jha M.K. (2011). Evaluating Hydrologic Response of an agricultural watershed for watershed analysis. Water.

[55] Yen H., Lu S., Feng Q., Wang R., Gao J., Brady D.M., Sharifi A., Ahn J., Chen S.T., Jeong J. (2017). Assessment of Optional Sediment Transport Functions via the Complex Watershed Simulation Model SWAT. Water.

[56] Brönnimann C.S. (2011). Effect of Groundwater on Landslide Triggering. Ph.D. Thesis.

[57] Premchitt J., Brand E.W., Phillipson H.B. (1986). Landslides caused by rapid groundwater changes. Geol. Soc. Lond. Eng. Geol. Pub..

[58] He H.S., DeZonia B.E., Mladenoff D.J. (2000). An aggregation index (AI) to quantify spatial patterns of landscapes. Landsc. Ecol..

[59] Jaeger J.A.G. (2000). Landscape division, splitting index, and effective mesh size: New measures of landscape fragmentation. Landsc. Ecol..

[60] Gergel S.E. (2005). Spatial and non-spatial factors: When do they affect landscape indicators of watershed loading?. Landsc. Ecol..

[61] Forman R.T.T. (1995). Land Mosaics: The Ecology of Landscape and Regions.

[62] Fahrig L. (2002). Effect of habitat fragmentation on the extinction threshold: A synthesis. Ecol. Appl..

[63] Kennedy C.M. (2013). A global quantitative synthesis of local and landscape effects on wild bee pollinators in agroecosystems. Ecol. Lett..

[64] Qiu J., Turner M.G. (2015). Importance of landscape heterogeneity in sustaining hydrologic ecosystem services in an agricultural watershed. Ecosphere.

[65] Yan B., Fang N.F., Zhang P.C., Shi Z.H. (2013). Impacts of land use change on watershed streamflow and sediment yield: An assessment using hydrologic modelling and partial least squares regression. J. Hydrol..

